# Editorial: Advancements in biomechanical modeling of injuries, diseases, diagnoses, and treatments of lower extremities

**DOI:** 10.3389/fbioe.2023.1178336

**Published:** 2023-04-11

**Authors:** Mohammad Nikkhoo, Lizhen Wang, Ching-Chi Hsu, Chih-Hsiu Cheng

**Affiliations:** ^1^ School of Physical Therapy and Graduate Institute of Rehabilitation Science, College of Medicine, Chang Gung University, Taoyuan, Taiwan; ^2^ Bone and Joint Research Center, Chang Gung Memorial Hospital, Linkou, Taiwan; ^3^ Department of Biomedical Engineering, Science and Research Branch, Islamic Azad University, Tehran, Iran; ^4^ School of Biological Science and Medical Engineering, Beihang University, Beijing, China; ^5^ Department of Mechanical Engineering, National Taiwan University of Science and Technology, Taipei, Taiwan

**Keywords:** biomechanics of lower extremities, mathematical musculoskeletal modeling, finite element modeling, hip biomechanics, knee biomechanics, leg injuries, footwear

The musculoskeletal system is a complex multi-articular structure which provides a relative range of motion for functional activities. It seems similarities between the joint biomechanics of the upper and the lower extremities. However, the upper extremity is more specialized for daily activities with greater ranges of motion, and the lower extremity structures are well prepared for locomotion and its weight-bearing (Yan et al., 2015). The basic principles of lower limb biomechanics focus on understanding the mechanical response and performance, the effect of injuries or diseases, and movement optimization in hope of finding effective rehabilitation procedures. Nevertheless, there is still a lack of knowledge in this research field, particularly at the level of injury risk factors, muscle functions, control, and instability. Hence, studying the biomechanical response of healthy, diseased, and treated lower extremities has been of great interest to clinicians and researchers. Numerous experimental, theoretical, as well as computational biomechanical studies have been conducted in the hope of understanding the underlying mechanisms involved in lower limb biomechanics. While some studies have been geared toward the prevention of injuries, others deal with more effective diagnostic and treatment modalities. Experimental clinical studies, using both *in-vitro* and *in-vivo* platforms, have the ability to provide relevant information regarding the biomechanical behavior and response of the lower extremities. Incidentally, these studies are essential but could be costly and limited by their inability to estimate the detailed microstructural phenomenon.

In the past few decades, different mathematical and computational methodologies of modeling [such as musculoskeletal and finite element (FE) modeling] have emerged as powerful and practical tools for non-invasive biomechanical investigations. These modeling approaches can explore the various aspects of the lower extremities, from the response of the health conditions in different movements, to evaluations of the effects of different pathologies and injuries, and to relevant treatment modalities and techniques. The current Research Topic presents a collection of cutting-edge studies which enhance our knowledge about biomechanical investigations of lower extremities and stimulate deliberations for the improvement of research techniques used for the development of relevant studies for injuries, diseases, diagnoses, and treatments.

Three studies focused on investigating the kinematics and kinetics of the whole lower extremity. Li et al. have quantified the kinetic and kinematic features of pedestrian active and avoidance behaviors in dangerous impact scenarios and pedestrian-vehicle interaction in the pre-crash phase using immersive virtual reality technology. They confirmed that the pedestrian kinetics and kinematics at the instant of occurrence of a collision were significantly influenced by their active avoidance behavior. In addition, the physical conditions, sufficient time, and a safe distance are required for effective avoidance motion. This study provided comprehensive experimental data which can be utilized as a valuable reference for developing and validating the FE models and multi-body simulations for lower limbs. Herrera-Valenzuela et al. have used a novel mathematical methodology behind the Gait Deviation Index (GDI) proposed by Schwartz and Rozumalski (2008) to a dataset of adults with spinal cord injury (SCI). Using the motion capture system on the lower limb, this novel index is calculated using 21-feature vectorial basis derived from gait data of the population with SCI. They have reported that implementation of the original GDI in population with SCI may result in an overestimation of gait function, but the new proposed index (SCI-GDI) could be more sensitive to larger gait impairment than the GDI. Prasad et al. proposed a simulation-based generalized framework for modelling and evaluation of cable-driven mobile lower limb exoskeleton is proposed which can be used for rehabilitation procedures. Simulation of the conceptual configurations in this study indicated that the 4-cable configuration is an encouraging design option for a lower limb rehabilitation exoskeleton based on tracking performance, component forces exerted on the lower limb, and motor power requirements.

During routine activities of daily living, the hip joint plays a crucial role in the mobility and stability of the lower extremities as the primary link between the upper and lower limbs. Hence, investigation of the hip biomechanics principles can be beneficial for the enhancement of the knowledge of the pathological mechanisms, diagnosis, and treatment techniques (Polkowski and Clohisy, 2010). Jiang et al. conducted a retrospective investigation for patients with femoral neck fractures (FNF) to comprehensively study the effect of vertical and oblique inclinations on fracture stability and reoperation risks using an innovative multivariant model. It was shown that fracture inclination in vertical and oblique planes is closely related to reoperation outcomes and evaluating the fracture stability using this methodology may be a beneficial complement to the classic Pauwels technique (van Embden et al., 2011). Chen et al. used the FE modeling to study the influence of anterior center-edge angle (ACEA) on stress distribution of the hip joint, which could enhance the clinical diagnosis of the severity of hip dysplasia. This study showed that in the patients with lower ACEA, the area ratio of high stress increased. They concluded that for cases with borderline developmental dysplasia of the hip, both the ACEA and the area of facies lunata played critical roles in characterizing the severity of hip dysplasia. Vega et al. used a patient-specific neuro-musculoskeletal model and direct collocation optimal control to estimate the impact of ipsilateral psoas muscle strength on gait cycle following internal hemipelvectomy surgery with prosthesis reconstruction. The results showed that when post-surgery psoas strength was increased, stance width and stride length returned to pre-surgery values. Hence, this study suggests that retention and strengthening of the psoas muscle on the operated side may be vital for maximizing post-surgery post-surgery gait function. Alexander et al. developed personalized musculoskeletal models to study lower limb kinematics and kinetics during gait in pediatric and adolescent patients with increased, isolated femoral anteversion compared to typically developing controls. The contribution of this study was to identify the specific subgroups of patients who may have a higher risk of joint overloading as a consequence of both altered morphology and kinematics. In conclusion, it was shown that the altered femoral morphology does not always result in an increased risk of joint overloading. However, personalized kinematics should be considered for better judgment.

Five studies focused on the pathological mechanism and surgical techniques for the treatment of fractures in Femur and Tibia. Cen et al. developed multiscale FE models of the proximal femur for both the elderly and young population to evaluate the mechanical responses at the tissue and cell scales in the mid-stance of gait. Mechanical responses of cortical bone and osteocytes in different quadrants of the mid-femoral neck were studied in the mid-stance of gait. It was confirmed that the mass and bone mineral density of femoral neck cortical bone could be correlated to the mechanical response of osteocytes. This could be valuable to adopt novel treatment techniques based on the improvement of cortical bone quality by stimulating osteocytes. In addition, quadrantal differences in bone quality in the mid-femoral neck might be deliberated to improve fracture risk prediction in future investigations. Wang et al. used a combination of the experimental study, topological optimization analyses, and FE modeling to evaluate the performance of a novel strategy for a minimally invasive plate osteosynthesis of distal tibia fractures. They showed that this technique which is called bionic lightweight design plating. It was shown that the application of FE simulation analyses combined with a topology optimization algorithm could improve the capacity of soft tissue protection by reducing the volume of the implant. They concluded that a smaller plate volume results in decreasing the excessive mechanical stimulation of soft tissues caused by the implants. Three of these investigations were focused on tibial plateau fractures (TPF) which are among the most common fractures in knee trauma. Ren et al. developed a novel plate through an anterolateral approach for posterolateral TPF and assessed the biomechanical performance of six different groups by the biomechanical experimental testing and FE simulations. This study concluded that the proposed patented plate had a proper biomechanical advantage for posterolateral TPF including balanced stress distribution which can reduce the risk of fixation failure. Moufid et al. measured the mechanical effects of cement injection on adjacent bone structures for stabilizing the TPF using the combination of the *in-vitro* experimental studies and FE analyses. They concluded that cement can provide proper reduction and primary stabilization, permitting the patient to undergo rehabilitation with active and passive mobilization. However, it is suggested that no weight-bearing would be authorized while the cortical bone is not stabilized. Vendeuvre et al. performed *in-vitro* experiments to comprehensively compare different minimally invasive fixations in association with balloon reduction based on the tuberoplasty method. They evaluated the primary structural strength of the TPF fixation regarding the separation and depression components and tested the mechanical influence of injectable bone cement filling on three different types of percutaneous screws. It was determined that cement filling during tuberoplasty could be a promising approach in association with percutaneous osteosynthesis to provide a better surgical outcome.

The knee, as the main lower limb motor joint, is comprising four bones and an extensive network of ligaments and muscles. Knee diseases, including musculoskeletal and neurological disorders, and the relevant treatment strategies may extremely affect the knee biomechanical response. Five studies were focused on knee biomechanics and the treatment modalities in this Research Topic. Guo et al. provided a systematic review and performed a meta-analysis on the impact of anterior cruciate ligament (ACL) defects and ACL integrity on clinical outcomes. They determined that no differences were observed in the primary clinical outcomes such as postoperative revision, and Tegner activity score and Oxford Knee Score in ACL deficient and unicondylar knee arthroplasty intact patients. Chen et al. performed experiments and developed a validated musculoskeletal model to evaluate the ACL biomechanics and the variation between the knee joint variables in three landing test actions. This study showed minimal differences in knee valgus, knee valgus moment, and ACL forces between the aforementioned landing actions. Nonetheless, it was reflected that knee flexion angle, knee extension moments sagittal factors, and quadriceps and gastrocnemius forces are the main risk factors for ACL injury. Cheng et al. developed a validated 3D FE model of a healthy cadaveric knee to find the optimal femoral drilling angle for ACL reconstruction with the anteromedial (AM) portal technique. It was presented that the femoral tunnel drilling angle could directly affect both the strain and stress distribution on the femoral tunnel, tibial tunnel, and ACL graft. A femoral tunnel drilling angle of 45° coronal/45° sagittal represented the lowest peak stress, maximum strain on the femoral and tibial tunnel entrance, and the lowest peak stress on the ACL graft. Ren et al. adapted a gait perturbation process induced the mechanism of human falls and quantified the lower limb response to examine the compensatory strategies of patients with knee osteoarthritis (KOA). It was found that patients with KOA have a higher risk of falling in response to a backward slip perturbation compared with healthy older individuals. Patients with KOA have a better focus on the strength and activation of the muscles that play a critical role in hip extension during gait. Lin et al. used a custom-made knee extension force measurement system to evaluate the effect of cycling training with a central fatigue challenge resistance on individuals with Parkinson’s disease (PD). This study confirmed that this training methodology could be beneficial, efficient, and feasible for patients with early-stage PD in strengthening both central and peripheral components of knee extensor forces and can be considered for developing rehabilitation interventions for these patients.

Studying foot and ankle biomechanics is another major field in lower extremities research investigations which focuses on the function of the foot and ankle in the normative, pathologic, and clinically treated states (Towers et al., 2003). Jiang et al. developed a multi-rigid body modeling technique to analyze the kinetics and kinematics of the lower limb in the half-squat parachuting landing to evaluate the protective effects of an ankle brace. It was found that this ankle brace might provide more effective protection for the lower-extremity joints in the half-squat parachuting landing with a backpack than no backpack landing. Zeng et al. developed a 3D fracture line mapping methodology to define the distribution of fracture lines throughout the ankle joint to describe a novel classification for the fibular and tibial fracture lines. They have classified 228 patients with ankle fractures using this methodology and claimed that this technique can include fracture types that cannot be identified by both AO and Lauge-Hansen (LH) classification. Rebelo et al. utilized a validated lower limb FE model of underbody blast (UBB) to estimate the effect of stature on injury risk. This study showed that there is a higher risk of calcaneal injury associated with short statures than medium and tall statures. In addition, they found that shorter individuals are at a higher risk of leg injury compared to taller ones. A combat boot and a floor mat were revealed to have a variable attenuating efficiency depending on the UBB loading characteristics. Funaro et al. developed a personalized FE model which contained the sub-tendons structure and their relative sliding to study the impact of rehabilitation maneuvers and different twists on Achilles tendon (AT) strains. It was shown that the rehabilitation maneuvers ranking was independent of the AT twist. However, the findings in this study could provide a better guide for clinicians to prescribe rehabilitation exercises for different twists on AT strains. Lv et al. utilized a 3D FE model of foot with a calcaneal fracture to assess the biomechanical performance of double-point fixation (DPF) compared to the three-point fixation technique. They concluded that DPF fixation by volar distal radius plates revealed favorable and sufficient fixation stability with a lower risk of postoperative stress fracture, which may possibly assist surgeons in planning a new fixation modality. Li et al. used a foot multi-segmental modeling to explore the kinematics and GRFs in marathon-experienced recreational runners before, during, and after long-distance running. It was found that excessive foot motion after 5 km of running may hypothetically increase the risks of running-related injuries (IRR). Moreover, the forefoot space of footwear may affect the biomechanical response of foot in long-distance running. In addition, Matias et al. studies the biomechanical-related risk factors for RRI and stated the effects of the 8-week foot-core exercise training program. They observed that changes in foot-joint kinematics resulting from the foot-core training program could be in charge of the reduction in RRI incidence. Xiao et al. measured the foot muscle strength, passive ankle kinesthesia, and postural control to examine the effects of 4 weeks of foot core exercise and multi-session high-definition transcranial direct current stimulation (HD-tDCS). The achieved results showed that HD-tDCS might increase the metatarsophalangeal joint flexor strength and the passive kinesthesia thresholds of ankle inversion and eversion. Footwear is generally designed to provide protection, assist in motion control and stability, and treat musculoskeletal deformities and injuries (Barton et al., 2009). Henceforward, numerous investigations are dedicated to this field of lower extremity biomechanics. Chen et al. used a validated musculoskeletal model to identify the immediate effects of worn shoes on the kinematics and kinetics of the lower limb. The outcomes of this research suggested that aging shoes are not appropriate. Attrition in the heel can raise the balance risk. Attrition shoes may affect the gait pattern and result in discomfort. Yu et al. studied the effects of heel-to-toe drops (HTD) and running speed on the biomechanical response of lower limb biomechanics using the inverse kinematics algorithm. This investigation showed that shoes with −8 mm HTD possibly will have a better ability to store and return energy that was related to running performance. Wang et al. combined the FE simulations with musculoskeletal modeling to study the effect of heel height on the strain distribution of plantar fascia. The results from the present research could reveal the strain variation on the plantar fascia and facilitate understanding of potential causes of plantar fasciitis induced by using high-heel shoes. In addition, this study claimed that the findings found that heel elevation as a treatment recommendation for plantar fasciitis could be questionable. Peng et al. used a detailed flatfoot orthosis FE model combined with Taguchi methodology to calculate the effect of design combinations on the response of foot-ankle biomechanics. It was shown that the foot orthosis with higher arch support and medial inclination angle could be more effective in reducing the peak plantar pressure. Besides, adding the medial forefoot posting may modify the forefoot deformity and forefoot pressure. Zhang et al. and Su et al. used a high-speed dual fluoroscopic imaging system to investigate the alterations of the first metatarsophalangeal joint (MTPJ) and medial longitudinal arch’s (MLA) kinematics between shod and barefoot running. Zhang et al. determined that shoe-wearing could limit the extension and joint translation of the first MTPJ and increase the horizontal adduction angle. This conclusion revealed that shoemakers would increase the capacity of the forefoot movement to reduce the injury risk factors. Su et al. presented that shoe-wearing may limit the partial movements of the MLA which may affect the storage and release of elastic energy during running.

Collectively, 31 peer-reviewed papers presented in this Research Topic tackle different challenges in the topic of biomechanical investigations of injuries, diseases, diagnoses, and treatments of lower extremities. The outputs of these studies could be generally utilized to either understand the causes of musculoskeletal system disorders, predict the risk factors, or enhance the effectiveness of certain treatment management strategies. Every study in this Research Topic reports its potential and existing limitations of the established technique and highlights the progress in its relevant research area. Hence, we believe that the current Research Topic could be fundamental to enhance understanding of lower limb biomechanics which may be used for clinical applications. However, various studies and clinical observations may need to be considered in the future to see these methodologies used routinely. As guest editors, and contributors, we hope this Research Topic can serve as state-of-the-art research investigations which may be beneficial for the design and development of new and more effective interventions for lower limb disorders.

## References

[B1] Barton C. J. Bonanno D. Menz H. B. (2009). Development and evaluation of a tool for the assessment of footwear characteristics. J. Foot Ankle Res. 2, 10. 10.1186/1757-1146-2-10 19389229 PMC2678108

[B2] Polkowski G. G. Clohisy J. C. (2010). Hip biomechanics. Sports Med. Arthrosc. Rev. 18 (2), 56–62. 10.1097/JSA.0b013e3181dc5774 20473123

[B3] Schwartz M. H. Rozumalski A. (2008). The gait deviation index: A new comprehensive index of gait pathology. Gait Posture 28 (3), 351–357. 10.1016/j.gaitpost.2008.05.001 18565753

[B4] Towers J. D. Deible C. T. Golla S. K. (2003). Foot and ankle biomechanics. Semin. Musculoskelet. Radiol. 7 (1), 67–74. 10.1055/s-2003-41086 12888945

[B5] van Embden D. Roukema G. R. Rhemrev S. J. Genelin F. Meylaerts S. A. (2011). The Pauwels classification for intracapsular hip fractures: Is it reliable? Injury 42 (11), 1238–1240. 10.1016/j.injury.2010.11.053 21146815

[B6] Yan T. Cempini M. Oddo C. M. Vitiello N. (2015). Review of assistive strategies in powered lower-limb orthoses and exoskeletons. Robotics Aut. Syst. 64, 120–136. 10.1016/j.robot.2014.09.032

